# Serotype 3 Remains the Leading Cause of Invasive Pneumococcal Disease in Adults in Portugal (2012–2014) Despite Continued Reductions in Other 13-Valent Conjugate Vaccine Serotypes

**DOI:** 10.3389/fmicb.2016.01616

**Published:** 2016-10-14

**Authors:** Andreia N. Horácio, Catarina Silva-Costa, Joana P. Lopes, Mário Ramirez, José Melo-Cristino, Portuguese Group for the Study of Streptococcal Infections, Teresa Vaz

**Affiliations:** Author Affiliations: Centro Hospitalar do Barlavento Algarvio; Hospital de Cascais; Centro Hospitalar de Coimbra; Centro Hospitalar de Entre Douro e Vouga; Centro Hospitalar de Lisboa Central; Centro Hospitalar Lisboa Norte; Centro Hospitalar Lisboa Ocidental; Centro Hospitalar de Vila Nova de Gaia/Espinho; Centro Hospitalar do Alto Ave; Centro Hospitalar do Baixo Alentejo; Centro Hospitalar do Porto; Centro Hospitalar da Póvoa do Varzim/Vila do Conde; Hospital de Vila Real; Hospitais da Universidade de Coimbra; Hospital Central do Funchal; Hospital Curry Cabral, Lisboa; Hospital de Santa Luzia, Elvas; Hospital de Santo André, Leiria; Hospital de São João, Porto; Hospital de Braga; Hospital Dr. José Maria Grande, Portalegre; Hospital do Espírito Santo, Évora; Hospital dos SAMS, Lisboa; Hospital Dr. Fernando da Fonseca, Amadora/Sintra; Hospital Garcia de Orta, Almada; Hospital Infante D. Pedro, Aveiro; Hospital de São Teotónio,Viseu; Hospital Pedro Hispano, Matosinhos; Instituto Nacional de Saúde Ricardo Jorge, Porto; Hospital Reynaldo dos Santos, Vila Franca de Xira; Unidade Local de Saúde do Alto Minho; Hospital CUF Descobertas; Centro Hospitalar do Tâmega e Sousa; Hospital Beatriz Ângelo, Loures; Centro Hospitalar de Setúbal; Hospital Distrital de Santarém; Centro Hospitalar do Médio Ave; Hospital de Faro; Centro Hospitalar do Oeste Norte; Hospital da Luz; Hospital da Figueira da Foz.; Faculty of Medicine, Instituto de Microbiologia, Instituto de Medicina Molecular, University of LisbonLisbon, Portugal

**Keywords:** *Streptococcus pneumoniae*, conjugate vaccines, polysaccharide vaccine, antimicrobial resistance, invasive disease, serotype

## Abstract

Since 2010 the 13-valent pneumococcal conjugate vaccine (PCV13) replaced the 7-valent vaccine (PCV7) as the leading pneumococcal vaccine used in children through the private sector. Although, neither of the PCVs were used significantly in adults, changes in adult invasive pneumococcal disease (IPD) were expected due to herd protection. We characterized *n* = 1163 isolates recovered from IPD in adults in 2012–2014 with the goal of documenting possible changes in serotype prevalence and antimicrobial resistance. Among the 54 different serotypes detected, the most frequent, accounting for half of all IPD, were serotypes: 3 (14%), 8 (11%), 19A (7%), 22F (7%), 14 (6%), and 7F (5%). The proportion of IPD caused by PCV7 serotypes remained stable during the study period (14%), but was smaller than in the previous period (19% in 2009–2011, *p* = 0.003). The proportion of IPD caused by PCV13 serotypes decreased from 51% in 2012 to 38% in 2014 (*p* < 0.001), mainly due to decreases in serotypes 7F and 19A. However, PCV13 serotype 3 remained relatively stable and the most frequent cause of adult IPD. Non-PCV13 serotypes continued the increase initiated in the late post-PCV7 period, with serotypes 8 and 22F being the most important emerging serotypes. Serotype 15A increased in 2012–2014 (0.7% to 3.5%, *p* = 0.011) and was strongly associated with antimicrobial resistance. However, the decreases in resistant isolates among serotypes 14 and 19A led to an overall decrease in penicillin non-susceptibility (from 17 to 13%, *p* = 0.174) and erythromycin resistance (from 19 to 13%, *p* = 0.034). Introduction of PCV13 in the NIP for children, as well as its availability for adults may further alter the serotypes causing IPD in adults in Portugal and lead to changes in the proportion of resistant isolates.

## Introduction

Two types of pneumococcal vaccines are licensed to prevent invasive pneumococcal disease (IPD), both targeting a restricted number of serotypes out of the 94 serotypes currently recognized in *Streptococcus pneumoniae*: strictly polysaccharide based vaccines and polysaccharide-protein conjugate based vaccines (PCVs) (Ramirez, 2014). The first licensed pneumococcal conjugate vaccine was the 7-valent pneumococcal conjugate vaccine (PCV7), which targets serotypes 4, 6B, 9V, 14, 18C, 19F, and 23F. PCV7 became available for children in the USA in 2000 and in Europe in 2001. Two additional conjugate vaccines became available more recently: a 10-valent vaccine (PCV10), which includes PCV7 serotypes and serotypes 1, 5, and 7F; and a 13-valent vaccine (PCV13), which includes PCV10 serotypes and serotypes 3, 6A, and 19A. PCVs proved to be highly effective in reducing the number of IPD episodes caused by vaccine serotypes (Pilishvili et al., 2010; Aguiar et al., 2014). Moreover, a decrease in IPD caused by PCV serotypes was also noted in non-vaccinated individuals (a phenomenon termed herd protection) (Horácio et al., 2013; Moore et al., 2015). However, use of PCVs was also accompanied by replacement of vaccine serotypes by non-vaccine types (NVTs) as causes of IPD, both in vaccinated children and in non-vaccinated adults. The overall impact of this phenomenon varied greatly around the world (Pérez-Trallero et al., 2009; Guevara et al., 2014; Harboe et al., 2014; Moore et al., 2015; Waight et al., 2015). The switch to the higher valency vaccines PCV10 and PCV13 also affected emerging serotypes. For instance, serotypes 7F and 19A were reported as emerging in IPD in the post-PCV7 period (Aguiar et al., 2010; Steens et al., 2013; Guevara et al., 2014; Harboe et al., 2014; Waight et al., 2015) but several studies have already shown that they decrease following PCV13 use (Aguiar et al., 2014; Moore et al., 2015; Waight et al., 2015). A 23-valent strictly polysaccharide vaccine (PPV23) includes 12 of the serotypes found in PCV13 (except 6A) and serotypes 2, 8, 9N, 10A, 11A, 12F, 15B, 17F, 20, 22F, and 33F. This vaccine has been used for two decades in older children and adults and has proven efficacy in the prevention of IPD (Moberley et al., 2013).

PCV7, PCV10 and PCV13 became available in Portugal in late-2001, mid-2009 and in early-2010, respectively. However, in contrast to many European countries, in Portugal PCV7 was not included in the national immunization program (NIP) and the uptake of PCV7 in children increased gradually over time, reaching 75% in 2008 (Aguiar et al., 2008). PCV13 replaced PCV7 since its availability and has been the most widely used pneumococcal vaccine since then, with estimates of 63% coverage in 2012 (Aguiar et al., 2014). PCV13 received an indication for adults ≥50 years in 2012 and in 2013 its indication was extended to all ages, but use of these vaccines in adults in Portugal was believed to be low until 2014. PCV13 was introduced into the NIP for children in 2015, being given free of charge to all children born from January 2015 onwards, with a 2+1 schedule (Direcção Geral de Saúde, 2015b). PPV23 is also available in Portugal since 1996, but its uptake among adults is estimated to be low (~10%) (Horácio et al., 2012). Since 2015, guidelines from the national health authorities recommend vaccinating adults in particular risk groups with both PCV13 and PPV23 (Direcção Geral de Saúde, 2015a). However, these groups will constitute a minority of the overall population and there are no guidelines recommending vaccinating adults more broadly with any of the pneumococcal vaccines.

In spite of the gradual increase in PCV uptake in children and the relatively modest coverage, we found significant changes in serotype distribution and antimicrobial susceptibility of pneumococci causing adult IPD that could be attributed at least in part to herd protection. The proportion of adult IPD caused by PCV13 serotypes was highest in 2008 (70%), but a gradual decrease took place until 2011, when only 54% of the isolates causing adult IPD expressed PCV13 serotypes (Horácio et al., 2012, 2013). In the present study we continued monitoring potential changes in serotype distribution and antimicrobial susceptibility of isolates causing adult IPD after PCV13 received an adult indication and before the introduction of PCV13 in the NIP for children.

## Materials and methods

### Ethics statement

Case reporting and isolate collection were considered to be surveillance activities and were exempt from evaluation by the Review Board of the Faculdade de Medicina of Universidade de Lisboa. The data and isolates were de-identified so that these were irretrievably unlinked to an identifiable person.

### Bacterial isolates

Invasive pneumococcal infections have been monitored in Portugal since 1999 by the Portuguese Group for the Study of Streptococcal Infections (Serrano et al., 2004). This is a laboratory-based surveillance system, in which 31 microbiology laboratories throughout Portugal are asked to identify all isolates responsible for IPD and to send them to a central laboratory for characterization. Although, the laboratories were contacted periodically to submit the isolates to the central laboratory, no audit was performed to ensure compliance, which may be variable in this type of study. A case of IPD was defined by the isolation of pneumococci from a normally sterile fluid, such as blood, pleural fluid or cerebral spinal fluid (CSF). The isolates included in the study were recovered from adult patients (≥18 years) with IPD between January 2012 and December 2014. Only one isolate from each patient in each year was included in the study. All isolates were identified as pneumococci by colony morphology, hemolysis on blood agar plates, optochin susceptibility and bile solubility.

### Serotyping and antimicrobial susceptibility testing

Serotypes were determined by the standard capsular reaction test using the chessboard system and specific sera (Sørensen, 1993) (Statens Serum Institut, Copenhagen, Denmark). Serotypes were classified into vaccine serotypes, i.e., those included in PCV7 (serotypes 4, 6B, 9V, 14, 18C, 19F, 23F), in PCV10 (all PCV7 serotypes plus serotypes 1, 5, and 7F), in PCV13 (all PCV10 serotypes plus 3, 6A, and 19A) or in PPV23 (all PCV13 serotypes, except serotype 6A and serotypes 2, 8, 9N, 10A, 11A, 12F, 15B, 17F, 20, 22F, and 33F) and non-vaccine serotypes (NVT). Given the high frequency of spontaneous switching between serotypes 15B and 15C we have opted to group isolates with these serotypes into a single group. Due to difficulties in phenotypically distinguishing isolates of serotype 25A and serogroup 38 these were also grouped together into the 25A/38.

Minimal inhibitory concentrations (MICs) for penicillin and cefotaxime were determined using Etest strips (Biomérieux, Marcy l'Étoile, France). In 2008, the CLSI changed the recommended breakpoints used to interpret MIC values. Unless otherwise stated we have used the CLSI-recommended breakpoints prior to 2008 (Clinical and Laboratory Standards Institute, 2007) as epidemiological breakpoints that allow the comparison with previous studies. Isolates were further characterized by determining their susceptibility to erythromycin, clindamycin, vancomycin, linezolid, tetracycline, levofloxacin, trimethroprim-sulfamethoxazole and chloramphenicol by the Kirby-Bauer disk diffusion technique, according to the CLSI recommendations and interpretative criteria (Clinical and Laboratory Standards Institute, 2014).

Macrolide resistance phenotypes were identified using a double disc test with erythromycin and clindamycin, as previously described (Melo-Cristino et al., 2003). Simultaneous resistance to erythromycin and clindamycin defines the MLS_B_ phenotype (resistance to macrolides, lincosamides and streptogramin B) while non-susceptibility only to erythromycin indicates the M phenotype.

### Statistical analysis

Simpson's index of diversity (SID) and respective 95% confidence intervals (CI95%) was used to measure the population diversity (Carriço et al., 2006). Adjusted Wallace (AW) coefficients were used to compare two sets of partitions (Severiano et al., 2011). These indices were calculated using the online tool available at http://www.comparingpartitions.info. Differences were evaluated by the Fisher exact test and the Cochran-Armitage test (CA) was used for trends with the false discovery rate (FDR) correction for multiple testing (Benjamini and Hochberg, 1995). A *p* < 0.05 was considered significant for all tests.

## Results

### Isolate collection

A total of 1163 isolates were collected from adults with invasive pneumococcal disease between 2012 and 2014: 404 in 2012, 383 in 2013 and 376 in 2014. The majority were recovered from blood (*n* = 1066, 91.7%) and the remaining from CSF (*n* = 59, 5.1%), pleural fluid (*n* = 26, 2.2%), peritoneal fluid (*n* = 9, 0.8%) and other normally sterile sites (*n* = 3, 0.3%).

### Serotype distribution

**Figure 1:**
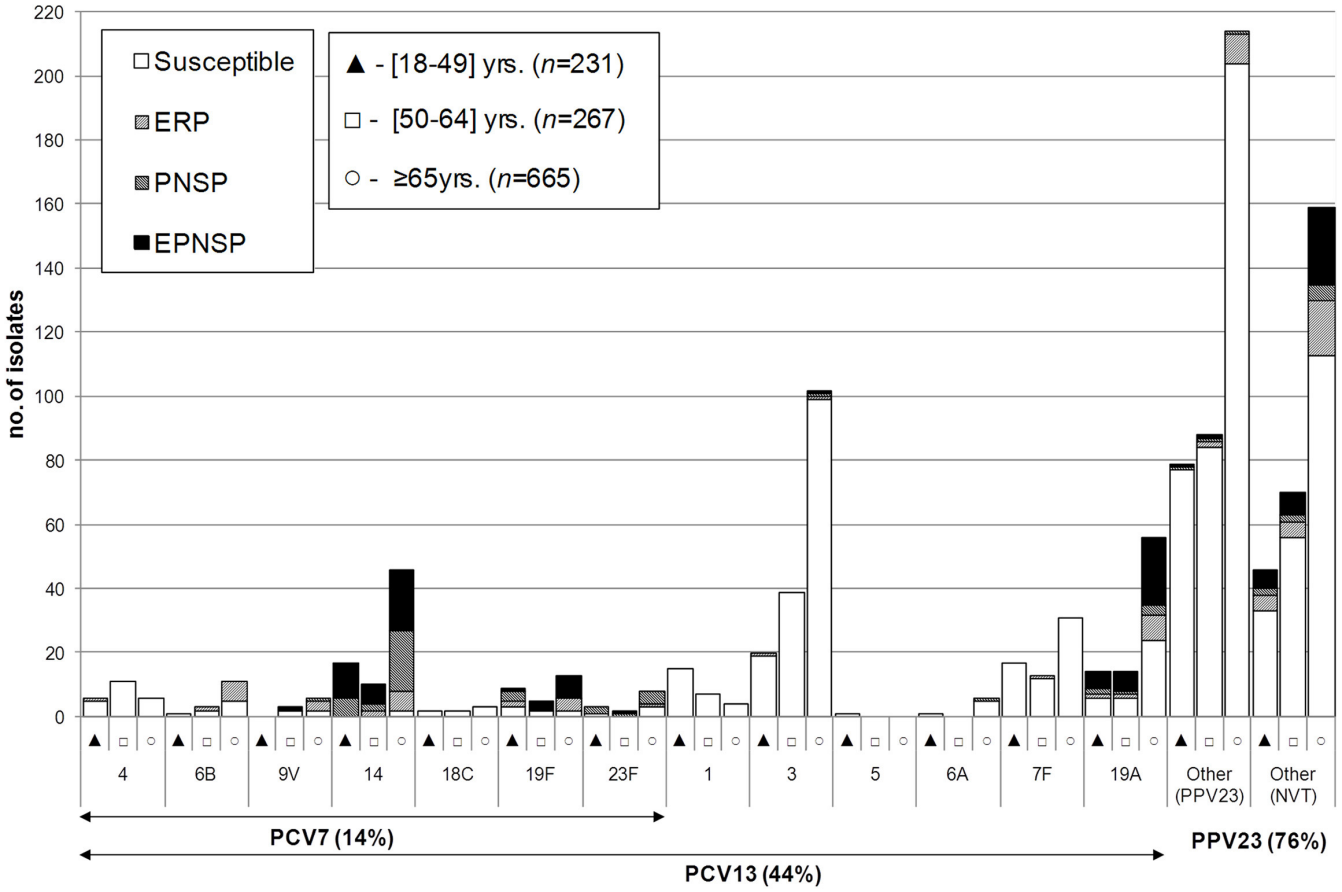
**Serotypes of isolates causing invasive pneumococcal disease in adult patients (≥18 years) in Portugal, 2012–2014**. The number of isolates expressing each serotype in each of the age groups considered is indicated. Isolates recovered from patients 18–49 years are indicated by black triangles, from patients 50–64 years by open squares, and from patients ≥65 years by open circles. Isolates presenting both erythromycin resistance and penicillin non-susceptibility (EPNSP) are represented by black bars. Penicillin non-susceptible isolates (PNSP) are indicated by dark hatched bars. Erythromycin resistant pneumococci (ERP) are indicated by light hatched bars. Isolates susceptible to both penicillin and erythromycin are represented by white bars. The serotypes included in the seven-valent conjugate vaccine (PCV7) and in the 13-valent conjugate vaccine (PCV13) are indicated by the arrows. NVT, non-vaccine serotypes; PPV23, 23-valent polysaccharide vaccine.

**Figure 2:**
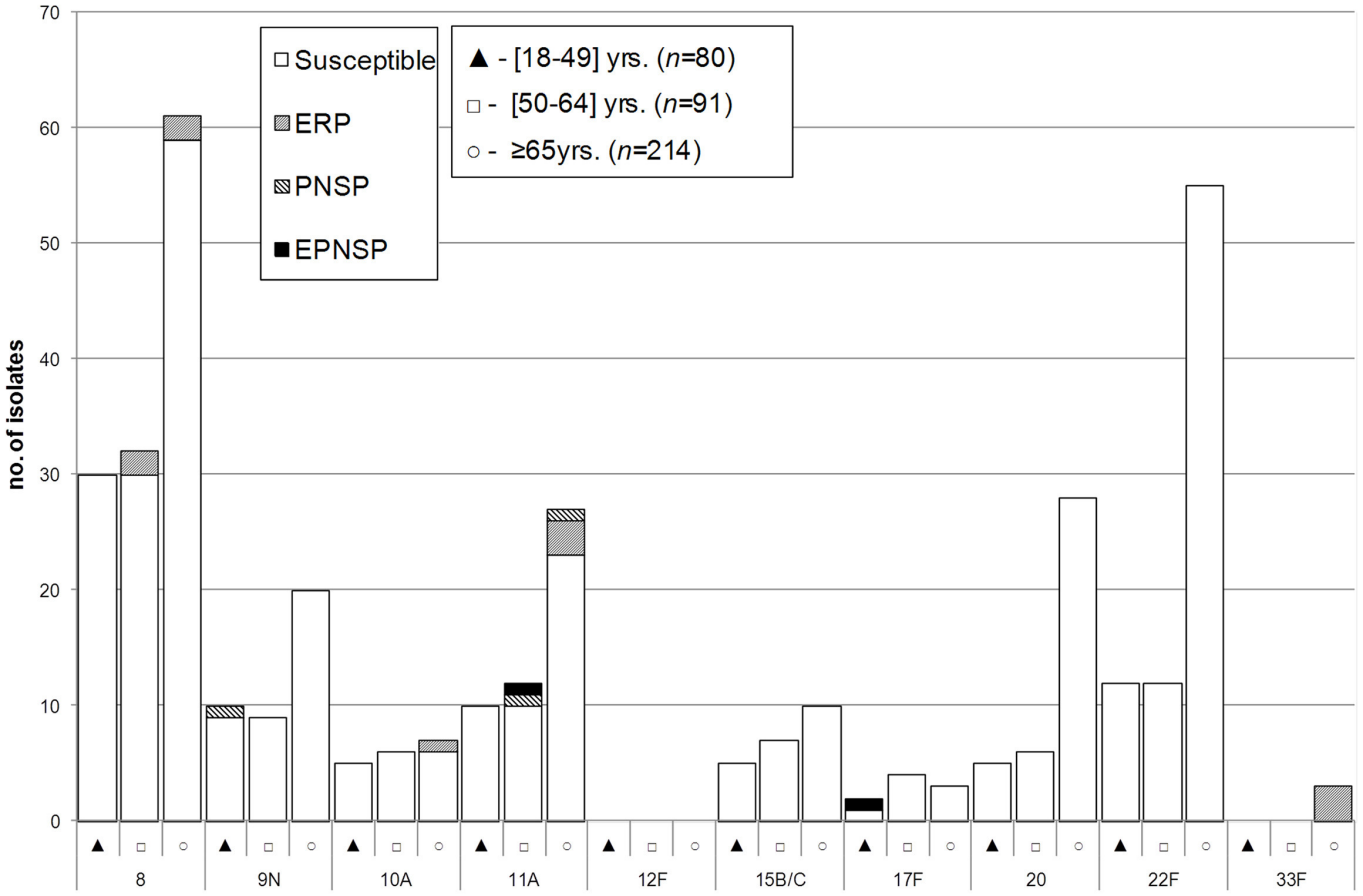
**Isolates expressing serotypes present in PPV23 but not included in conjugate vaccines causing invasive pneumococcal disease in adult patients (≥18 years) in Portugal, 2012–2014**. See legend of Figure 1. Out of the 11 serotypes present in PPV23 but absent from PCV13, serotype 2 was not found in our collection.

**Figure 3:**
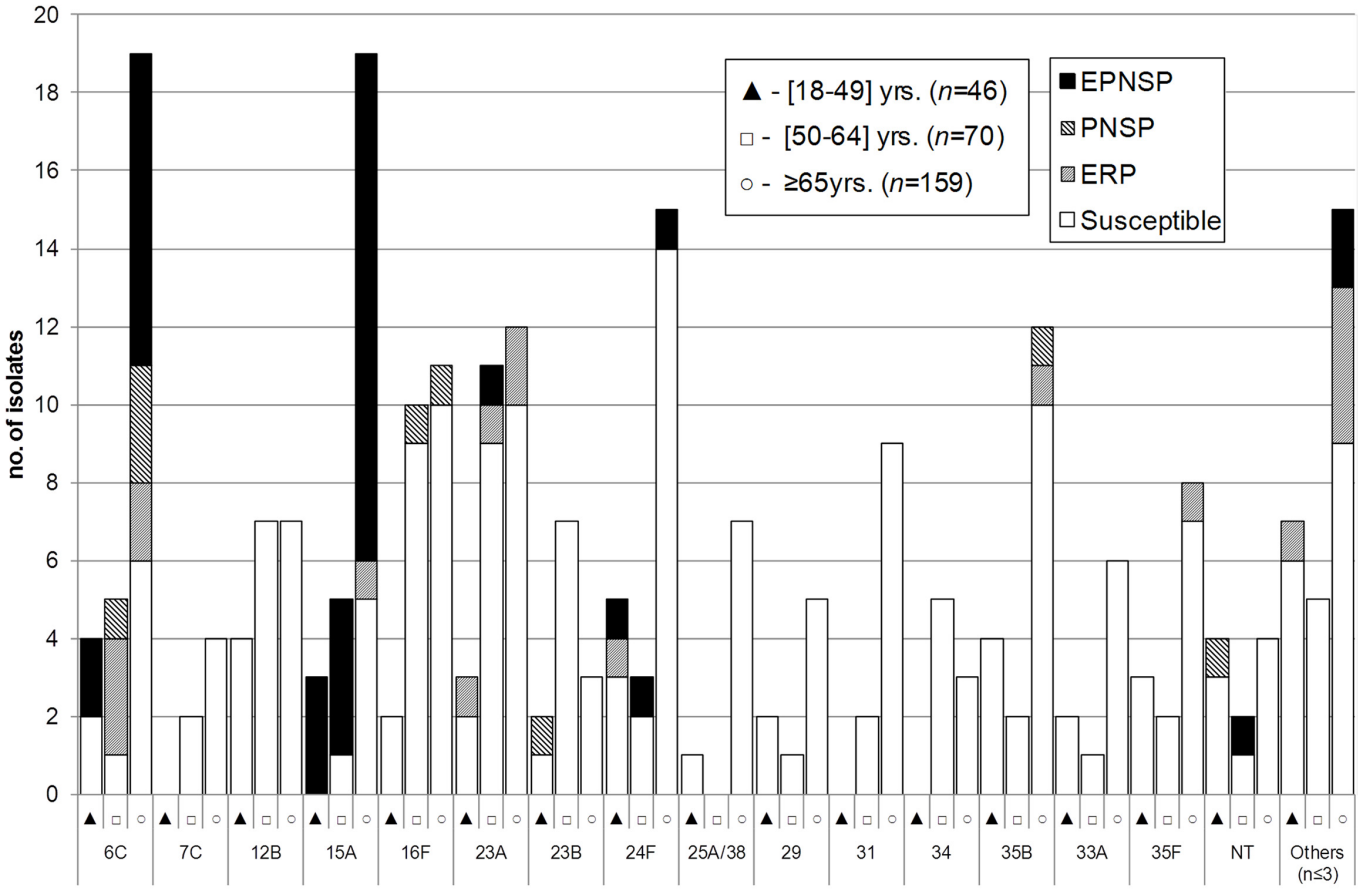
**Isolates expressing serotypes not included in any pneumococcal vaccine causing invasive pneumococcal disease in adult patients (≥18 years) in Portugal, 2012–2014**. See legend of Figure 1. NT, non-typable. Isolates expressing serotype 25A and 38 could not be distinguished phenotypically and are represented together. Only serotypes including *n* > 3 isolates are discriminated.

Between 2012 and 2014, a total of 54 different serotypes were identified. The most frequent, which accounted for half of the isolates were serotypes 3 (*n* = 161, 13.8%), 8 (*n* = 123, 10.6%), 19A (*n* = 84, 7.2%), 22F (*n* = 79, 6.8%), 14 (*n* = 73, 6.3%), and 7F (*n* = 61, 5.2%). Figures 1–3 represent the number of isolates expressing serotypes included in PCVs, the additional serotypes found in PPV23, and the number of isolates expressing NVTs stratified by age group. Serotype diversity was high (2012–2014 SID = 0.944, CI95%: 0.939–0.949). Although, diversity was >0.93 in all the studied years, there was a small but significant increase in serotype diversity between 2012 (SID = 0.935, CI95%: 0.924–0.945) and 2013 (SID = 0.950, CI95%: 0.942–0.958) (*p* = 0.019).

Serotype distribution varied according to age group but serotype diversity was not different in the three age groups considered (18–49 years, SID = 0.948, CI95%: 0.938–0.958; 50–64 years, SID = 0.945, CI95%: 0.933–0.957; ≥65 years, SID = 0.939, CI95%: 0.931–0.946). Only for serotype 1 were the differences in age distribution statistically supported after FDR correction with the proportion of serotype 1 decreasing with age (accounting for 6.5, 2.6, and 0.6% of the isolates recovered from patients aged 18–49 years, 50–64 years and ≥65 years, respectively, CA *p* < 0.001). In contrast, the proportion of IPD caused by the group of additional serotypes found only in PCV13 (3, 6A, and 19A) increases with age (15.2% in 18–49 years, 19.9% in 50–64 years and 24.7% in ≥65 years, CA *p* = 0.002, significant after FDR).

When considering serotypes presenting three or more CSF isolates, we found a positive association with CSF for serotypes 19F (*p* = 0.006) and 23B (*p* = 0.005), both significant after FDR correction (Table S1). No significant associations with serotype were found for isolates recovered from pleural fluid.

**Figure 4:**
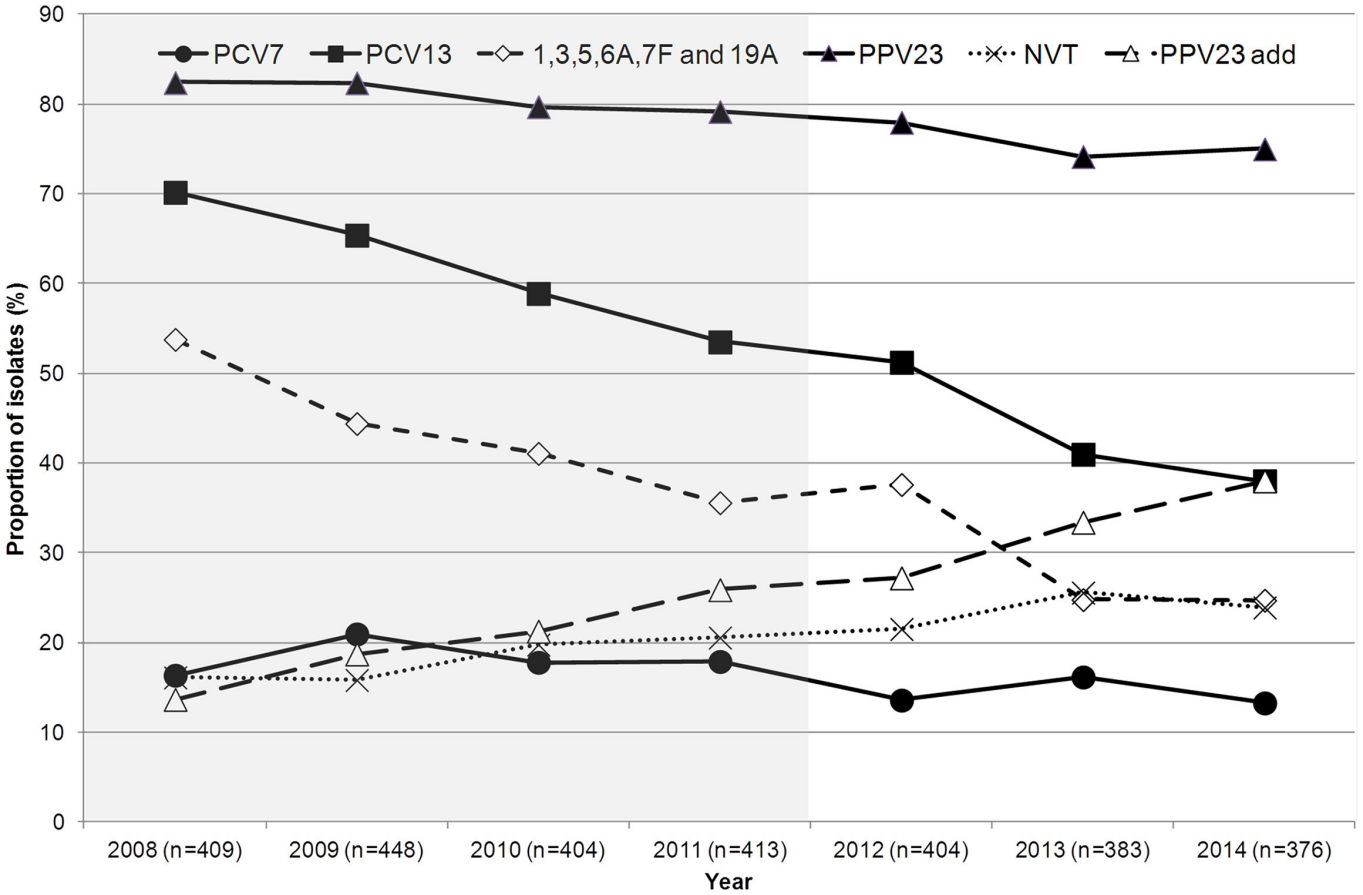
**Proportion of isolates expressing serotypes included in pneumococcal vaccines causing invasive pneumococcal disease in adult patients (≥18 years) in Portugal, 2008–2014**. The data up to 2011 were presented previously (Horácio et al., 2012, 2013).

Figure 4 shows the proportion of potentially vaccine preventable IPD during the study period and, for comparison purposes, also from 2008 to 2011 since important changes in serotype distribution initiated in this period (Horácio et al., 2013). Considering the current study period only (2012–2014), the overall proportion of IPD caused by PCV7 serotypes remained stable, while there was a decrease in the proportion of IPD caused by the additional serotypes found in both PCV10 and PCV13 (serotypes 1, 5, 7F; from 11.1 to 4.8%, *p* = 0.001, significant after FDR) and in PCV13 only (serotypes 3, 6A, and 19A; from 26.5 to 19.9%, *p* = 0.024, significant after FDR). This resulted in the overall decrease in the proportion of IPD caused by PCV13 serotypes from 51.2% in 2012 to 38.0% in 2014 (*p* < 0.001, significant after FDR). The proportion of IPD caused by PPV23 serotypes and NVTs did not suffer significant changes during the study period (Figure 4). However, the proportion of IPD caused by the additional serotypes found only in PPV23 (PPV23 add) significantly increased, from 27.2 to 38.0% (*p* = 0.001, significant after FDR). When considering the evolution of potentially vaccine preventable IPD in the entire period from 2008 to 2014, there was a decrease in the overall proportion of IPD caused by PCV13 serotypes, although this was temporarily interrupted in 2012, mainly due to a slight increase of serotype 3 (see below).

Table 1 shows the evolution of individual serotypes causing adult IPD from 2008 to 2014. When looking for trends in the proportion of individual serotypes during the current study period (2012–2014), the only significant change that was supported after FDR correction was the decrease in serotype 7F (from 8.2% in 2012 to 4.7% in 2013 and 2.7% in 2014, CA *p* < 0.001). No significant changes in the proportion of individual serotypes were detected during the study period when stratifying by age group (data not shown).

**Table 1:** **Serotypes of the isolates responsible for invasive pneumococcal disease in adult patients (≥18 years), 2008–2014**.

**Serotype**	**No. of isolates (%)**							**CA^a^**	**CA**
					**Current study period**				
	**2008**	**2009**	**2010**	**2011**	**2012**	**2013**	**2014**	**2012–2014**	**2008–2014**
**PCV13**									
1	55 (13.4)	48 (10.7)	22 (5.4)	17 (4.1)	12 (3.0)	7 (1.8)	7 (1.9)	0.289	<**0.001**
3	51 (12.5)	53 (11.8)	59 (14.6)	48 (11.6)	66 (16.3)	45 (11.7)	50 (13.3)	0.209	0.613
4	10 (2.4)	12 (2.7)	17 (4.2)	14 (3.4)	6 (1.5)	8 (2.1)	9 (2.4)	0.361	0.352
5	12 (2.9)	9 (2.0)	4 (1.0)	0 (0)	0 (0)	0 (0)	1 (0.3)	0.211	<**0.001**
6A	6 (1.5)	8 (1.8)	2 (0.5)	1 (0.2)	2 (0.5)	1 (0.3)	4 (1.1)	0.315	0.062
6B	1 (0.2)	7 (1.6)	3 (0.7)	9 (2.2)	5 (1.2)	5 (1.3)	5 (1.3)	0.909	0.272
7F	48 (11.7)	48 (10.7)	35 (8.7)	43 (10.4)	33 (8.2)	18 (4.7)	10 (2.7)	**0.001**	<**0.001**
9V	14 (3.4)	7 (1.6)	8 (2.0)	5 (1.2)	4 (1.0)	4 (1.0)	1 (0.3)	0.255	<**0.001**
14	29 (7.1)	45 (10.0)	30 (7.4)	31 (7.5)	29 (7.2)	26 (6.8)	18 (4.8)	0.172	0.045
18C	0 (0)	6 (1.3)	1 (0.2)	1 (0.2)	1 (0.2)	4 (1.0)	2 (0.5)	0.588	0.675
19A	48 (11.7)	33 (7.4)	44 (10.9)	38 (9.2)	39 (9.7)	24 (6.3)	21 (5.6)	0.027	0.005
19F	7 (1.7)	13 (2.9)	8 (2.0)	5 (1.2)	9 (2.2)	12 (3.1)	6 (1.6)	0.576	0.956
23F	6 (1.5)	4 (0.9)	5 (1.2)	9 (2.2)	1 (0.2)	3 (0.8)	9 (2.4)	0.005	0.618
**PPV23 add**									
8	15 (3.7)	19 (4.2)	27 (6.7)	33 (8.0)	34 (8.4)	43 (11.2)	46 (12.2)	0.081	<**0.001**
9N	10 (2.4)	12 (2.7)	13 (3.2)	11 (2.7)	8 (2.0)	13 (3.4)	18 (4.8)	0.030	0.122
10A	3 (0.7)	8 (1.8)	7 (1.7)	6 (1.5)	2 (0.5)	8 (2.1)	8 (2.1)	0.062	0.294
11A	7 (1.7)	13 (2.9)	10 (2.5)	16 (3.9)	16 (4.0)	18 (4.7)	15 (4.0)	0.974	0.012
12F	0 (0)	1 (0.2)	0 (0)	0 (0)	0 (0)	0 (0)	0 (0)	–	0.334
15B/C	8 (2.0)	4 (0.9)	3 (0.7)	8 (1.9)	5 (1.2)	9 (2.3)	8 (2.1)	0.353	0.096
17F	3 (0.7)	2 (0.4)	4 (1.0)	4 (1.0)	5 (1.2)	2 (0.5)	2 (0.5)	0.255	0.981
20	4 (1.0)	8 (1.8)	5 (1.2)	7 (1.7)	14 (3.5)	11 (2.9)	14 (3.7)	0.851	**0.001**
22F	10 (2.4)	17 (3.8)	16 (4.0)	22 (5.3)	25 (6.2)	23 (6.0)	31 (8.2)	0.261	<**0.001**
33F	0 (0)	0 (0)	1 (0.2)	0 (0)	1 (0.2)	1 (0.3)	1 (0.3)	0.959	0.180
**NVT**^b^									
6C	4 (1.0)	13 (2.9)	13 (3.2)	10 (2.4)	8 (2.0)	14 (3.7)	6 (1.6)	0.757	0.600
15A	4 (1.0)	5 (1.1)	5 (1.2)	5 (1.2)	3 (0.7)	11 (2.9)	13 (3.5)	0.011	**0.002**
23A	6 (1.5)	8 (1.8)	8 (2.0)	4 (1.0)	9 (2.2)	8 (2.1)	9 (2.4)	0.879	0.317
16F	3 (0.7)	8 (1.8)	3 (0.7)	7 (1.7)	13 (3.2)	3 (0.8)	7 (1.9)	0.161	0.233
24F	3 (0.7)	6 (1.3)	5 (1.2)	2 (0.5)	5 (1.2)	9 (2.3)	9 (2.4)	0.241	0.027
12B	10 (2.4)	5 (1.1)	3 (0.7)	11 (2.7)	6 (1.5)	8 (2.1)	4 (1.1)	0.649	0.700
35B	2 (0.5)	5 (1.1)	10 (2.5)	5 (1.2)	6 (1.5)	4 (1.0)	8 (2.1)	0.480	0.222
35F	2 (0.5)	3 (0.7)	2 (0.5)	3 (0.7)	7 (1.7)	4 (1.0)	2 (0.5)	0.110	0.350
23B	4 (1.0)	3 (0.7)	7 (1.7)	6 (1.5)	4 (1.0)	5 (1.3)	3 (0.8)	0.801	0.958
31	2 (0.5)	2 (0.4)	1 (0.2)	5 (1.2)	5 (1.2)	2 (0.5)	4 (1.6)	0.786	0.197
NT	0 (0)	3 (0.7)	4 (1.0)	3 (0.7)	1 (0.2)	3 (0.8)	6 (1.6)	0.042	0.060
33A	1 (0.2)	4 (0.9)	8 (2.0)	4 (1.0)	2 (0.5)	5 (1.3)	2 (0.5)	0.929	0.913
25A/38	2 (0.5)	3 (0.7)	0 (0)	2 (0.5)	3 (0.7)	3 (0.8)	2 (0.5)	0.726	0.583
29	0 (0)	0 (0)	0 (0)	0 (0)	4 (1.0)	2 (0.5)	2 (0.5)	0.433	0.009
34	2 (0.5)	0 (0)	0 (0)	8 (1.9)	3 (0.7)	1 (0.3)	4 (1.1)	0.605	0.138
7C	2 (0.5)	2 (0.4)	2 (0.5)	1 (0.2)	1 (0.2)	4 (1.0)	1 (0.3)	0.942	0.883
18A	6 (1.5)	0 (0)	1 (0.2)	2 (0.5)	0 (0)	3 (0.8)	0 (0)	0.959	0.080
21	3 (0.7)	0 (0)	0 (0)	4 (1.0)	0 (0)	0 (0)	0 (0)	–	0.108
Others^c^	8 (2.0)	1 (0.2)	8 (2.0)	3 (0.7)	7 (1.7)	9 (2.3)	8 (2.1)	–	–
Total	409	448	404	413	404	383	376	–	–

a *CA, Cochran Armitage test of trend. In bold are the serotypes with significant p-values (p < 0.05) after FDR correction*.

b *NVT, non-vaccine serotypes*.

c Only serotypes detected in ≥3 isolates in at least one year are shown; the remaining are represented in “Others.”

When considering together data from 2008 to 2014 there were changes (significant after FDR) in the proportion of individual serotypes. There were decreases in the proportion of IPD caused by serotypes: 1 (from 13.4 to 1.9%, CA *p* < 0.001), 5 (from 2.9 to 0.3%, CA *p* < 0.001), 9V (from 3.4 to 0.3%, CA *p* < 0.001) and 19A (from 11.7 to 5.6%, CA *p* = 0.005). In contrast, there were increases in the proportion of IPD caused by PPV23 serotypes: 8 (from 3.7 to 12.2%, CA *p* < 0.001), 22F (from 2.4 to 8.2%, CA *p* < 0.001) and 20 (from 1.0 to 3.7%, CA *p* = 0.001); and an increase of the NVT 15A (from 1.0 to 3.5%, CA *p* = 0.002). Even though these changes were statistically supported when analyzing data from 2008 to 2014, in the case of serotypes 19A and 15A, the more disparate values were only detected from 2013 onwards, while for serotype 20, this occurred from 2012 onwards.

Table 2 shows the evolution of IPD serotypes during the study period (2012–2014) according to vaccine serotypes and stratified by age group. Recapitulating what was seen when considering all age groups together (Figure 4), a decrease in the overall proportion of IPD caused by PCV13 serotypes was detected in the three age groups considered; however, only for individuals ≥65 years was this statistically supported (Table 2). Moreover, only for this age group was the decrease in the additional serotypes found in both PCV10 and PCV13 (serotypes 1, 5 and 7F) statistically supported after FDR correction (Table 2). When analyzing the evolution of each serotype from 2008 to 2014 stratifying by age group, only serotype 1 decreased in all age groups considered (CA *p* < 0.001 for each, significant after FDR correction), while the increase of serotype 8 was significant only in the two older groups (≥50 years) (CA *p* < 0.001 for both, significant after FDR correction), and the changes in serotypes 5, 7F, 19A, 20, and 22F were statistically supported only in individuals ≥65 years (CA *p* < 0.001 for serotypes 5 and 7F, CA *p* = 0.007 for serotype 19A, CA *p* = 0.003 for serotype 20 and CA *p* = 0.001 for serotype 22F, all significant after FDR correction).

**Table 2:** **Number of isolates responsible for invasive pneumococcal disease in adult patients (≥18 years), according to vaccine serotype groups and age groups, 2012–2014**.

	**Serotype groups**	**No. isolates (%)**			**CA^a^**
		**2012**	**2013**	**2014**	
18–49 years	PCV7^b^	18 (21.4)	12 (15.0)	8 (11.9)	0.112
	1, 5, and 7F	15 (17.9)	10 (12.5)	8 (11.9)	0.286
	3, 6A, and 19A	12 (14.3)	12 (15.0)	11 (16.4)	0.719
	PCV13^c^	45 (53.6)	34 (42.5)	27 (40.3)	0.094
	PPV23 add^d^	26 (31.0)	29 (36.3)	24 (35.8)	0.511
	NVTs^e^	13 (15.5)	17 (21.3)	16 (23.9)	0.191
50–64 years	PCV7^b^	7 (9.2)	14 (13.9)	15 (16.7)	0.164
	1, 5, and 7F	10 (13.2)	6 (5.9)	4 (4.4)	0.037
	3, 6A, and 19A	20 (26.3)	19 (18.8)	14 (15.6)	0.087
	PCV13^c^	37 (48.7)	39 (38.6)	33 (36.7)	0.124
	PPV23 add^d^	17 (22.4)	34 (33.7)	37 (41.1)	0.011
	NVTs^e^	22 (28.9)	28 (27.7)	20 (22.2)	0.316
≥65 years	PCV7^b^	30 (12.3)	36 (17.8)	27 (12.3)	0.947
	1, 5, and 7F	20 (8.2)	9 (4.5)	6 (2.7)	**0.008**
	3, 6A, and 19A	75 (30.7)	39 (19.3)	50 (22.8)	0.042
	PCV13^c^	125 (51.2)	84 (41.6)	83 (37.9)	0.004
	PPV23 add^d^	67 (27.5)	65 (32.2)	82 (37.4)	0.022
	NVTs^e^	52 (21.3)	53 (26.2)	54 (24.7)	0.384

a *CA, Cochran Armitage test of trend. In bold are the serotype groups with significant p-values (p < 0.05) after FDR correction*.

b *PCV7, serotypes included in the 7-valent pneumococcal conjugate vaccine*.

c *PCV13, serotypes included in the 13-valent pneumococcal conjugate vaccine*.

d *PPV23 add, the additional 11 serotypes present in the 23-valent pneumococcal polysaccharide vaccine but absent from the 13-valent pneumococcal conjugate vaccine*.

e *NVTs, serotypes not included in any of the currently available pneumococcal vaccines*.

### Antimicrobial susceptibility

Resistance to the antimicrobials tested is summarized in Table 3. A total of *n* = 179 isolates (15.4%) were classified as penicillin non-susceptible pneumococci (PNSP): *n* = 160 (89.4%) presenting low level resistance and *n* = 19 (10.6%), high level resistance. Considering current CLSI breakpoints for penicillin, *n* = 12/59 CSF isolates (20.3%) would have been considered resistant and only *n* = 5/1104 non-CSF isolates (0.5%) would have been considered intermediately resistant. A total of *n* = 198 isolates (17.0%) were classified as erythromycin resistant pneumococci (ERP). Of these, *n* = 159 presented the MLS_B_ phenotype, while the remaining (*n* = 39, 19.7%) presented the M phenotype. Isolates simultaneously non-susceptible to penicillin and erythromycin (EPNSP) accounted for 10.4% of the collection (*n* = 121).

**Table 3:** **Antimicrobial resistance of the isolates responsible for invasive pneumococcal disease in adult patients (≥18 years) in Portugal, 2012–2014**.

	**No. resistant isolates (%)**		
	**18–49 years (*n* = 231)**	**50–64 years (*n* = 267)**	**≥65 years (*n* = 665)**
PEN	40 (17.3)	32 (12.0)	107 (16.1)
MIC_90_	0.38	0.125	0.25
MIC_50_	0.016	0.012	0.016
CTX	4 (1.7)	3 (1.1)	6 (0.9)
MIC_90_	0.25	0.19	0.25
MIC_50_	0.016	0.016	0.016
LEV	0 (0)	1 (0.4)	6 (0.9)
ERY	34 (14.7)	37 (13.9)	127 (19.1)
CLI	31 (13.4)	30 (11.2)	100 (15.0)
CHL	5 (2.2)	6 (2.2)	8 (1.2)
SXT	30 (13.0)	39 (14.6)	93 (14.0)
TET	23 (10.0)	21 (7.9)	79 (11.9)

*PEN, penicillin; CTX, cefotaxime; LEV, levofloxacin; ERY, erythromycin; CLI, clindamycin; CHL, chloramphenicol; SXT, trimethoprim/sulphamethoxazole; TET, tetracycline. All isolates were susceptible to vancomycin and linezolid*.

Antimicrobial resistance did not change significantly between age groups. In 2012–2014, there was a significant decrease in antimicrobial resistance for several antimicrobials—erythromycin resistance decreased from 18.8 to 13.0% (CA *p* = 0.034), clindamycin resistance decreased from 16.1 to 10.4% (CA *p* = 0.022) and tetracycline resistance decreased from 13.4 to 7.7% (CA *p* = 0.010). Although, not statistically supported, there was also a decrease in penicillin non-susceptibility, from 16.8% in 2012 to 13.3% in 2014 (CA *p* = 0.174).

There was some correlation between serotype and antimicrobial resistance (Figures 1–3). The AW for serotype and PNSP was 0.569 (CI95%: 0.507–0.631) and the AW for serotype and ERP was 0.527 (CI95%: 0.458–0.596). Serotypes 14 and 19A were the most frequent serotypes among PNSP and ERP. Serotype 14 accounted for 35.2% of PNSP and 22.2% of ERP while serotype 19A occurred in 21.2% of PNSP and 21.2% of ERP. Taken together, PCV7 serotypes accounted for 48.6% of PNSP, 37.9% of ERP and 40.5% of EPNSP. Considering the PCV13 serotypes, these constituted 71.5, 61.1, and 67.8% of PNSP, ERP and EPNSP, respectively. The additional serotypes found in PPV23 but not in PCV13 accounted for only 2.8, 6.6, and 1.7% of PNSP, ERP and EPNSP, respectively. The proportion of resistant isolates was higher among isolates expressing NVTs: 25.7, 32.3, and 30.6% of PNSP, ERP and EPNSP, respectively (Figures 1–3). The most frequent NVTs among PNSP and ERP were serotypes 6C and 15A, which together accounted for 19.0% of PNSP and 18.2% of ERP (Figure 3).

## Discussion

The decrease in PCV13 serotypes observed previously (Horácio et al., 2012, 2013) continued during the present study period resulting in only 38.0% of the isolates collected in 2014 expressing PCV13 serotypes (Figure 4). However, different serotypes underlie the changes in 2008–2011 and 2012–2014.

The timeframes of the decreases seen for serotypes 7F and 19A are consistent with a possible herd protection of childhood vaccination with the most recently introduced PCVs. Similar decreases in serotypes 7F and 19A as causes of adult IPD followed the use of PCV13 in children in the USA (Moore et al., 2015) and in several European countries (Steens et al., 2013; Guevara et al., 2014; Harboe et al., 2014; Waight et al., 2015). Decreases in the incidence of IPD caused by these two serotypes were also documented among children in Portugal (Aguiar et al., 2014). In Portugal the decrease in serotype 7F preceded that of serotype 19A in adult IPD. This could have been attributed to the use of PCV10 in children, since PCV10 includes serotype 7F but not serotype 19A. Moreover, this vaccine was introduced in Portugal months earlier than PCV13. However, in children, serotype 19A decreased as a cause of IPD before an effect of PCV13 was expected and before any decrease in serotype 7F (Aguiar et al., 2014). This points to the importance of other factors besides vaccination in triggering changes in serotype prevalence and suggest that the initial changes seen in serotype 7F IPD in adults are the result of secular trends.

In contrast to these serotypes, there was no overall reduction of serotype 3. These results are concordant with other studies that failed to show a consistent reduction of serotype 3 among adult IPD after the use of PCV13 in children (Steens et al., 2013; Harboe et al., 2014; Moore et al., 2015; Waight et al., 2015) and with a study that demonstrated a low and non-significant effectiveness of PCV13 against serotype 3 IPD in children (Andrews et al., 2014).

The proposed higher efficacy of PCV13 against serotype 19F (Dagan et al., 2013) cannot explain the decrease in proportion of PCV7 serotypes, since serotype 19F was uncommon in our collection and no significant decrease was seen between the two periods (Table 1). The reduction of the overall proportion of IPD caused by PCV7 serotypes was instead related with decreases in serotypes 4, 9V and 14 (Table 1). Among these, serotype 4 exhibited the most significant decrease. Since the most significant decrease of serotype 14 IPD was detected in 2014, it remains uncertain if it will be sustained in the following years. Serotype 14 has been the most frequent PCV7 serotype causing adult IPD in Portugal, both before and after PCV7 use in children. This could be associated with particular characteristics of the highly successful and resistant clone Spain^14^-ST156, to which this serotype was found to be associated (Horácio et al., 2016). High antimicrobial consumption in our country could also contribute significantly to maintain resistant clones such as this one in circulation.

The non-PCV serotypes that increased the most since the late-post PCV7 period were those found in PPV23, especially serotypes 8, 22F, and 20 (ranked by frequency); but also the non-PPV23 serotype 15A (Table 1). Serotypes 15A and 22F were found in carriage in adults in Portugal (Almeida et al., 2014), while serotypes 8 and 20 were not found in carriage in adults and were shown to have a high invasive disease potential (Sá-Leão et al., 2011). Serotype 8 was the second most frequent cause of IPD during the current study period and in 2013 and 2014 was the most frequent cause of IPD among younger adults (18–49 years). Serotype 8 increased in importance as a cause of IPD in other countries, being the most frequent cause of IPD in patients aged >5 years in England and Wales after the introduction of PCV13 (Waight et al., 2015) and also important in adult IPD elsewhere (Guevara et al., 2014; Regev-Yochay et al., 2015). Serotype 22F became the second most frequent cause of IPD in adults aged ≥65 years in 2013 and 2014. In the USA, this serotype was the most common cause of adult IPD in the post-PCV13 period (Moore et al., 2015). An increase of serotype 22F after PCV13 use was also reported in Canada (Demczuk et al., 2013) and in some European countries (Steens et al., 2013; Lepoutre et al., 2015). Serotype 20 increased more modestly and only among individuals aged ≥65 years. An increase of this serotype was also noted in Canada, although mostly among individuals aged 15–49 years (Demczuk et al., 2013). Taken together, these observations indicate that, although there may be some regional differences, there are serotypes that seem to be consistently emerging in different geographic locations in the post-PCV13 period. These may reflect circulating serotypes in asymptomatic carriers but also serotypes with an enhanced invasive disease potential.

In 2014, the last year of the study, serotype 15A surpassed serotype 19A and 14 to become the most frequent serotype among ERP and was the second most frequent serotype among PNSP behind serotype 14. The overall decreases observed in PNSP and ERP were not only due to decreases in the total number of isolates expressing serotypes 14 and 19A, which were not compensated by the increase in serotype 15A (Table 1), but also to an unexpected decrease in the proportion of resistant isolates within serotypes 14 and 19A. While 72% of serotype 14 and 64% of serotype 19A were ERP in 2012, only 44% of serotype 14 and 33% of serotype 19A were ERP in 2014 (*p* = 0.071 and *p* = 0.031, respectively). Similarly, there was a decrease in the proportion of PNSP among serotype 19A, from 59% in 2012 to 24% in 2014 (*p* = 0.014).

Our surveillance system is exclusively laboratory based and lacks compliance audits, so our study was not designed to estimate the incidence of adult IPD. However, we did note a slight decrease in the number of isolates sent to us in 2013 and 2014 (Figure 4). This could reflect a net reduction of adult IPD following PCV13 use in children, as reported by others (Guevara et al., 2014; Harboe et al., 2014; Lepoutre et al., 2015; Moore et al., 2015; Regev-Yochay et al., 2015) and seen with IPD in children in Portugal (Aguiar et al., 2014). Alternatively, this could reflect lower reporting by participating laboratories. We also noted a marked decrease in the number of isolates recovered from younger patients relative to either of the older age groups when comparing 2009–2011 to 2012–2014 (*p* < 0.001) (Figure 1) (Horácio et al., 2013). Even if the decrease in number of isolates is attributed to lower reporting, we have no reason to believe that this would affect preferentially a particular age group. We also have no indication of changes in clinical practice (such as blood culturing practices), which could influence these results. We therefore believe that the most likely explanation is a true reduction in incidence of IPD in 18–49 years old individuals, in agreement with a study from the UK that found that this group was the one where the decrease in IPD incidence was more pronounced and followed more closely PCV13 use in children (Waight et al., 2015).

As discussed above, our study was not designed to allow the estimate of the incidence of IPD and it therefore does not evaluate potential changes in incidence with time. Specifically, although we include the majority of medical centers in Portugal our surveillance is not comprehensive and we did not perform audits to ensure that participating centers reported all cases, namely we did not include cases for which no viable pneumococcal isolate was received for characterization. However, the design based on the reporting of all isolates causing IPD within the surveillance network, the large number of isolates studied, the wide coverage of the country by the network and the stable number of reporting centers, guarantees that the data accurately represents IPD in Portugal and can be used to evaluate changes in the relative importance of the different serotypes.

In spite of relatively modest vaccine coverage (63% in 2012), there were major changes in the serotype distribution of the pneumococcal population responsible for adult IPD in Portugal following the use of PCVs in children consistent with herd protection. These changes have contributed also to significant reductions in antimicrobial resistance. The recent inclusion of PCV13 in the NIP for children in Portugal may have an even greater impact on IPD in adults. This remarkable effect of PCVs in protecting non-vaccinated individuals may question the need of using PCV13 directly in vaccinating adults. Still, data from 2014 indicates that the overall proportion of adult IPD caused by PCV13 serotypes remained significant (38%) and that isolates expressing PPV23 serotypes accounted for 75% of all IPD. Taken together this suggests a key role of vaccination in any effective management strategy of IPD.

## Author contributions

JM and MR: Conceived and designed the experiments. PGSSI: Collected data. AH, CS, and JL: Performed the experiments. AH, JM, and MR: Analyzed the data. All authors contributed to the writing of the manuscript and approved the version to be submitted.

### Conflict of interest statement

JM has received research grants administered through his university and received honoraria for serving on the speakers bureaus of Pfizer, Bial, GlaxoSmithKline and Novartis. MR has received honoraria for serving on speakers bureau of Pfizer and for consulting for GlaxoSmithKline. The other authors declare that the research was conducted in the absence of any commercial or financial relationships that could be construed as a potential conflict of interest.

## References

[B1] Aguiar S. I. Brito M. Horácio A. N. Lopes J. Ramirez M. Melo-Cristino J.. (2014). Decreasing incidence and changes in serotype distribution of invasive pneumococcal disease in persons aged under 18 years since introduction of 10-valent and 13-valent conjugate vaccines in Portugal, July 2008 to June 2012. Euro Surveill. 19:20750. 10.2807/1560-7917.ES2014.19.12.20750 24698140

[B2] Aguiar S. I. Pinto F. R. Nunes S. Serrano I. Melo-Cristino J. Sá-Leão R.. (2010). Denmark^14^-230 clone as an increasing cause of pneumococcal infection in Portugal within a background of diverse serotype 19A lineages. J. Clin. Microbiol. 48, 101–108. 10.1128/JCM.00665-09 19864476 PMC2812288

[B3] Aguiar S. I. Serrano I. Pinto F. R. Melo-Cristino J. Ramirez M. (2008). Changes in *Streptococcus pneumoniae* serotypes causing invasive disease with non-universal vaccination coverage of the seven-valent conjugate vaccine. Clin. Microbiol. Infect. 14, 835–843. 10.1111/j.1469-0691.2008.02031.x 18844684

[B4] Almeida S. T. Nunes S. Santos Paulo A. C. Valadares I. Martins S. Breia F.. (2014). Low prevalence of pneumococcal carriage and high serotype and genotype diversity among adults over 60 years of age living in Portugal. PLoS ONE 9:e90974. 10.1371/journal.pone.0090974 24604030 PMC3946249

[B5] Andrews N. J. Waight P. A. Burbidge P. Pearce E. Roalfe L. Zancolli M.. (2014). Serotype-specific effectiveness and correlates of protection for the 13-valent pneumococcal conjugate vaccine: a postlicensure indirect cohort study. Lancet Infect. Dis. 14, 839–846. 10.1016/S1473-3099(14)70822-9 25042756

[B6] Benjamini Y. Hochberg Y. (1995). Controlling the false discovery rate – a practical and powerful approch to multiple testing. J. R. Stat. Soc. Ser. B. Methodol. 57, 289–300.

[B7] Carriço J. A. Silva-Costa C. Melo-Cristino J. Pinto F. R. de Lencastre H. Almeida J. S.. (2006). Illustration of a common framework for relating multiple typing methods by application to macrolide-resistant Streptococcus pyogenes. J. Clin. Microbiol. 44, 2524–2532. 10.1128/JCM.02536-05 16825375 PMC1489512

[B8] Clinical Laboratory Standards Institute (2007). Performance Standards for Antimicrobial Susceptibility Testing – Seventeenth Informational Supplement. Wayne, PA: Clinical and Laboratory Standards Institute.

[B9] Clinical Laboratory Standards Institute (2014). Performance Standards for Antimicrobial Susceptibility Testing – Twenty-Fourth Informational Supplement. Wayne, PA: Clinical and Laboratory Standards Institute.

[B10] Dagan R. Patterson S. Juergens C. Greenberg D. Givon-Lavi N. Porat N.. (2013). Comparative immunogenicity and efficacy of 13-valent and 7-valent pneumococcal conjugate vaccines in reducing nasopharyngeal colonization: a randomized double-blind trial. Clin. Infect. Dis. 57, 952–962. 10.1093/cid/cit428 23804191

[B11] Demczuk W. H. B. Martin I. Griffith A. Lefebvre B. McGeer A. Lovgren M.. (2013). Serotype distribution of invasive *Streptococcus pneumoniae* in Canada after the introduction of the 13-valent pneumococcal conjugate vaccine, 2010–2012. Can. J. Microbiol. 59, 778–788. 10.1139/cjm-2013-0614 24313450

[B12] Direcção Geral de Saúde (2015a). Norma 11/2015 -Vacinação Contra Infeções Por Streptococcus Pneumoniae de Grupos com Risco Acrescido Para Doença Invasiva Pneumocócica (DIP). Adultos (≥18 anos de idade).

[B13] Direcção Geral de Saúde (2015b). Norma 12/2015 -Vacinação Contra Infeções Por Streptococcus Pneumoniae de Grupos com Risco Acrescido Para Doença Invasiva Pneumocócica (DIP). Idade pediátrica (< 18 anos de idade).

[B14] Guevara M. Ezpeleta C. Gil-Setas A. Torroba L. Beristain X. Aguinaga A.. (2014). Reduced incidence of invasive pneumococcal disease after introduction of the 13-valent conjugate vaccine in Navarre, Spain, 2001–2013. Vaccine 32, 2553–2562. 10.1016/j.vaccine.2014.03.054 24674661

[B15] Harboe Z. B. Dalby T. Weinberger D. M. Benfield T. Mølbak K. Slotved H. C.. (2014). Impact of 13-valent pneumococcal conjugate vaccination in invasive pneumococcal disease incidence and mortality. Clin. Infect. Dis. 59, 1066–1073. 10.1093/cid/ciu524 25034421

[B16] Horácio A. N. Diamantino-Miranda J. Aguiar S. I. Ramirez M. Melo-Cristino J. the Portuguese Group for the Study of Streptococcal Infections (2012). Serotype changes in adult invasive pneumococcal infections in Portugal did not reduce the high fraction of potentially vaccine preventable infections. Vaccine 30, 218–224. 10.1016/j.vaccine.2011.11.022 22100892

[B17] Horácio A. N. Diamantino-Miranda J. Aguiar S. I. Ramirez M. Melo-Cristino J. the Portuguese Group for the Study of Streptococcal Infections (2013). The majority of adult pneumococcal invasive infections in Portugal are still potentially vaccine preventable in spite of significant declines of serotypes 1 and 5. PLoS ONE 8:e73704. 10.1371/journal.pone.0073704 24066064 PMC3774749

[B18] Horácio A. N. Silva-Costa C. Diamantino-Miranda J. Lopes J. P. Ramirez M. Melo-Cristino J.. (2016). Population structure of *Streptococcus pneumoniae* causing invasive disease in adults in Portugal before PCV13 availability for adults: 2008-2011. PLoS ONE 11:e0153602. 10.1371/journal.pone.0153602 27168156 PMC4864403

[B19] Lepoutre A. Varon E. Georges S. Dorléans F. Janoir C. Gutmann L.. (2015). Impact of the pneumococcal conjugate vaccines on invasive pneumococcal disease in France, 2001–2012. Vaccine 33, 359–366. 10.1016/j.vaccine.2014.11.011 25448105

[B20] Melo-Cristino J. Ramirez M. Serrano N. Hänscheid T. The Portuguese Surveillance Group for the Study of Respiratory Pathogens (2003). Macrolide resistance in *Streptococcus pneumoniae* isolated from patients with community-acquired lower respiratory tract infections in Portugal: results of a 3-year (1999–2001) multicenter surveillance study. Microb. Drug Resist. 9, 73–80. 10.1089/107662903764736364 12705685

[B21] Moberley S. Holden J. Tatham D. P. Andrews R. M. (2013). Vaccines for preventing pneumococcal infection in adults. Cochrane Database Syst. Rev. 1:CD000422. 10.1002/14651858.CD000422.pub3 23440780 PMC7045867

[B22] Moore M. R. Link-Gelles R. Schaffner W. Lynfield R. Lexau C. Bennett N. M.. (2015). Effect of use of 13-valent pneumococcal conjugate vaccine in children on invasive pneumococcal disease in children and adults in the USA: analysis of multisite, population-based surveillance. Lancet Infect. Dis. 15, 301–309. 10.1016/S1473-3099(14)71081-3 25656600 PMC4876855

[B23] Pérez-Trallero E. Marimon J. M. Ercibengoa M. Vicente D. Pérez-Yarza E. G. (2009). Invasive *Streptococcus pneumoniae* infections in children and older adults in the north of Spain before and after the introduction of the heptavalent pneumococcal conjugate vaccine. Eur. J. Clin. Microbiol. Infect. Dis. 28, 731–738. 10.1007/s10096-008-0693-1 19153783

[B24] Pilishvili T. Lexau C. Farley M. M. Hadler J. Harrison L. H. Bennett N. M.. (2010). Sustained reductions in invasive pneumococcal disease in the era of conjugate vaccine. J. Infect. Dis. 201, 32–41. 10.1086/648593 19947881

[B25] Ramirez M. (2014). *Streptococcus pneumoniae*, in Molecular Medical Microbiology, eds Tang Y. W. Sussman M. Liu D. Poxton I. Schwartzman J. (Amsterdam: Academic Press, Elsevier), 1529–1546.

[B26] Regev-Yochay G. Paran Y. Bishara J. Oren I. Chowers M. Tziba Y.. (2015). Early impact of PCV7/PCV13 sequential introduction to the national pediatric immunization plan, on adult invasive pneumococcal disease: A nationwide surveillance study. Vaccine 33, 1135–1142. 10.1016/j.vaccine.2015.01.030 25613717

[B27] Sá-Leão R. Pinto F. Aguiar S. Nunes S. Carriço J. A. Frazão N.. (2011). Analysis of invasiveness of pneumococcal serotypes and clones circulating in Portugal before widespread use of conjugate vaccines reveals heterogeneous behavior of clones expressing the same serotype. J. Clin. Microbiol. 49, 1369–1375. 10.1128/JCM.01763-10 21270219 PMC3122870

[B28] Serrano I. Ramirez M. The Portuguese Surveillance Group for the Study of Respiratory Pathogens, Melo-Cristino, J. (2004). Invasive *Streptococcus pneumoniae* from Portugal: implications for vaccination and antimicrobial therapy. Clin. Microbiol. Infect. 10, 652–656. 10.1111/j.1469-0691.2004.00869.x 15214879

[B29] Severiano A. Pinto F. R. Ramirez M. Carriço J. A. (2011). Adjusted Wallace coefficient as a measure of congruence between typing methods. J. Clin. Microbiol. 49, 3997–4000. 10.1128/JCM.00624-11 21918028 PMC3209087

[B30] Sørensen U. B. (1993). Typing of pneumococci by using 12 pooled antisera. J. Clin. Microbiol. 31, 2097–2100. 8370735 10.1128/jcm.31.8.2097-2100.1993 PMC265703

[B31] Steens A. Bergsaker M. A. R. Aaberge I. S. Rønning K. Vestrheim D. F. (2013). Prompt effect of replacing the 7-valent pneumococcal conjugate vaccine with the 13-valent vaccine on the epidemiology of invasive pneumococcal disease in Norway. Vaccine 31, 6232–6238. 10.1016/j.vaccine.2013.10.032 24176490

[B32] Waight P. A. Andrews N. J. Ladhani S. N. Sheppard C. L. Slack M. P. E. Miller E. (2015). Effect of the 13-valent pneumococcal conjugate vaccine on invasive pneumococcal disease in England and Wales 4 years after its introduction: an observational cohort study. Lancet Infect. Dis. 15, 535–543. 10.1016/S1473-3099(15)00028-6 25801458

