# Supplementary material for: Serotype 3 Remains the Leading Cause of Invasive Pneumococcal Disease in Adults in Portugal (2012–2014) Despite Continued Reductions in Other 13-Valent Conjugate Vaccine Serotypes
Source: Front Microbiol. 2016 Oct 14;7:1616. doi: 10.3389/fmicb.2016.01616 (PMC5064670; doi:10.3389/fmicb.2016.01616)
Supplement: Supplementary file 1 [file Table1.PDF]

Table S1: Capsular types of the isolates recovered from CSF in adult patients (≥18 yrs), Portugal, 2012-2014.

	No. Isolates		
Serotype	CSF	non-CSF	OR ($CI_{95\%}$)
3	12	153	1.16 (0.55-2.30)
**19F**	**6**	**21**	**4.41 (1.41-12.02)**
11A	4	45	1.30 (0.33-3.76)
**23B**	**4**	**8**	**7.60 (1.62-29.43)**
8	3	122	0.32 (0.06-1.01)
16F	3	21	2.11 (0.39-7.38)
24F	3	20	2.22 (0.41-7.81)
Others	24	458	0.58 (0.33-1.03)
